# Reconstructing Ancient Egyptian Diet through Bone Elemental Analysis Using LIBS (Qubbet el Hawa Cemetery)

**DOI:** 10.1155/2015/281056

**Published:** 2015-08-06

**Authors:** Ghada Darwish Al-Khafif, Rokia El-Banna

**Affiliations:** ^1^Anthropology and Mummy Conservation Laboratory, Conservation and Research Center, Ministry of Antiquities, 4 Nobar Street, Ismail Pasha Palace, Lazoghli, Cairo 11521, Egypt; ^2^Biological Anthropology Department, National Research Center, El Buhouth Street, Dokki, Cairo 12311, Egypt

## Abstract

One of the most important advantages of LIBS that make it suitable for the analysis of archeological materials is that it is a quasi-nondestructive technique. Archeological mandibles excavated from Qubbet el Hawa Cemetery, Aswan, were subjected to elemental analysis in order to reconstruct the dietary patterns of the middle class of the Aswan population throughout three successive eras: the First Intermediate Period (FIP), the Middle Kingdom (MK), and the Second Intermediate Period (SIP). The bone Sr/Ca and Ba/Ca ratios were significantly correlated, so the Sr/Ca ratios are considered to represent the ante-mortem values. It was suggested that the significantly low FIP Sr/Ca compared to that of both the MK and the SIP was attributed to the consumption of unusual sorts of food and imported cereals during years of famine, while the MK Sr/Ca was considered to represent the amelioration of climatic, social, economic, and political conditions in this era of state socialism. The SIP Sr/Ca, which is nearly the same as that of the MK, was considered to be the reflection of the continuity of the individualism respect and state socialism and a reflection of agriculture conditions amelioration under the reign of the 17th Dynasty in Upper Egypt.

## 1. Introduction

Generally, social, economic, and belief system of a society can be reflected in food [1]. Information about the ancient Egyptians diet is mainly provided by artistic and textual sources [2]. But it is important to note that there many difficulties that interrupt the precise identification of food types consumed in ancient Egypt such as problems of translation [3]. However, as calcified tissues as bones and teeth can contain the indicators of diet and the environmental conditions, they are considered as the biological “archives” of the living organisms [4]. Thus, elemental analysis of archeological bones can be used as an important tool for paleodiet reconstruction.

From the Neolithic era and throughout the historic era, the base of masses daily diet was cereal foods. Beside bread and beer, the ancient Egyptian meals were mainly set from vegetables, fruit, milk, dairy products, and fish. Also, many species of fattened poultry or wild birds were eaten in ancient Egypt. The regular consumption of beef is observed in the higher social class [5].

Dietary calcium ions may be accompanied by Sr and Ba ions that are removed through a food chain due to what is called “*biopurification*” which is defined by Burton [6] as “*the collection of processes that tend to preferentially remove these ions from calcium as it progress through the food chain from lower to higher consumers*.”

The intestinal absorption ratio for Ca, Sr, and Ba is 10 : 5 : 1, respectively [7]. Once strontium is absorbed, it will be distributed throughout the body but its deposition will be mostly in bone and teeth [8].

Many techniques of elemental analysis are used to evaluate the apatite elemental composition of archeological bone and teeth samples, for example, atomic absorption spectroscopy (AAS) [9–12], atomic emission spectroscopy (AES) [13–18], and neutron activation analysis (NAA) [19, 20].

Although Samek et al. [21] reported on the use of laser induced breakdown spectroscopy (LIBS) in the quantitative detection of trace elements in human teeth and bones by creation of calibration curves for aluminum, lead, and strontium, few studies applied LIBS technique in the elemental analysis of archeological skeletal remains, such as the studies of Alvira et al. [22, 23], El-Tayeb [24], Galiová et al. [25], and Kasem et al. [26]. While, as indicated by Giakoumaki et al. [27], one of the most important advantages of LIBS—that makes it suitable for archeological science applications is that it is nearly a non-invasive method of analysis as there is no sample preparation, in addition, destruction caused by the ablation of tens to hundred nanograms from the target surface is microscopic.

The aim of the current study is to reconstruct the paleodiet of the Elephantine nobles followers and descendants (the middle class of the archeological Aswan population) through three successive historical eras: the First Intermediate Period (FIP), the Middle Kingdom (MK), and the Second Intermediate Period (SIP) using LIBS technique for the elemental analysis of mandibular bones.

## 2. Materials and Methods

### 2.1. Materials

Qubbet el Hawa is the cemetery of nobles of “Abu” (Elephantine), the first Upper Egyptian Nome capital. The cemetery consists of a large number of graves cut into the sand stone of the eastern mountain slope of the Nile west bank of Aswan. The human remains content of the graves are not only that of noble families, but also that of followers. The social rank was estimated according to the position of the corpus within the grave: the nuclear noble family was buried in the central grave chambers, while in shaft fillings and in the grave chambers followers or later descendants (middle class) were buried [28, 29]. The excavated bones were stored in the magazine grave number 30 on the site [30].

69 mandibles of the archeological site Qubbet el Hawa consist of the sample of the current work. The mandibles under investigation, which are stored now in the Anthropology and Mummy Conservation Lab., Ministry of Antiquities, belong to the archeological period including the FIP (7th−11th early Dynasties), the MK (the second part of the 11th-12th Dynasties) and the SIP (13th–17th Dynasties) as indicated in Table 1.

Only mandibles of adult individuals of the middle class were used in the current study.

Two soil samples were extracted from inside the archeological bones: one for elemental analysis and the other for pH measurement.

### 2.2. Methods

Soft brushes were used for a dry mechanical cleaning; then ethanol was applied on cotton buds to ensure a complete removal of dust and soil particles as directed by Hillson [31].

The eruption of the third molar is considered as an indication of an age exceeding 18 years [32]. The soil sample pH was measured using a calibrated portable pH meter (METTLER TOLEDO, Seven GoTM, pH meter SG2, Electrode: In Lab Surface).

All elemental determinations were carried out in the Laser Atomic Spectroscopy Laboratory (I) of the Department of Laser Applications in Metrology, Photochemistry and Agriculture (LAMPA), The National Institute of Laser Enhanced Sciences (NILES), Cairo University.

Laser-induced plasma was obtained using Q-switched Nd:YAG laser (Brio, Quantel, France) operating at its fundamental wavelength (*λ* = 1064 nm). The laser pulse energy was 100 mJ with a pulse duration 5 ns. Laser light was focused onto the target surface using a lens of focal length 10 cm. The collected plasma emission was transmitted through the optical fiber to the echelle spectrometer coupled to the coupled to the ICCD camera. The gate and the delay time are controlled through a personal computer that is used also for displaying and storing the obtained spectra. The gate time and the delay time were adjusted at 1000 ns for each. All measurements were performed in air at atmospheric pressure. Two laser pulses were applied to the desired position for surface cleaning. Bone analysis was performed by irradiating each mandible at 2 different positions on the surface of the cortical bone of the mandibular body, each by 5 laser pulses. The 10 obtained spectra were averaged.

Prior to elemental analysis, soil sample was prepared to form a pellet. The soil was finely grounded; then it was pressed using a hydraulic piston without using any sort of fillers. The elemental analysis of the soil sample was carried out by shooting 4 different positions on the surface of the soil pellet, each by 5 laser pulses. The 20 obtained spectra were averaged.

Finally, the analysis of the emission spectra was performed using the LIPS++ software. To minimize the effect of experimental parameters fluctuations, emission line of carbon at 2478 Å was used for normalization of relative intensities of all spectral lines.

A data base of collected qualitative and quantitative data was created using Microsoft Office Excel 2007 (Microsoft).

Sr calibration curve created by Samek et al. [21] was used to obtain quantitative data. Samek et al. [21] used artificial reference samples of calcium CaCO_3_ with known amount of SrCO_3_ (10–10000 ppm relative to Ca content of the matrix). Relative intensities of strontium were obtained at 461 nm and that of calcium at 432 nm. The line equation and *r* ^2^ value is obtained after El-Tayeb [24].

Sr/Ca ratio and Ba/Ca ratios were transformed into the logarithmic form as Burton et al. [33] stated that “*The log-transform creates a more normal distribution than the strongly positively skewed ppm data and obviates some of the bias of anomalous outliers*.”

The statistical analysis was performed using the SPSS statistics software (version 17) (IBM).

## 3. Results

### 3.1. Comparison between Soil and Bone Elemental Relative Intensities

The soil pH measured for Qubbet el Hawa Cemetery was 6.6 (i.e., neutral). The elemental analysis of bone and soil samples using LIBS was performed: Ca was detected at 432 nm, Sr at 461 nm, and Ba at 455.5 nm. The emission line intensity (a.u.) of an element represents the relative concentration of this element in the sample. The mean and the standard deviation were calculated as shown in Table 2.

### 3.2. Correlation between Bone Ba/Ca and Sr/Ca

**Figure 1:**
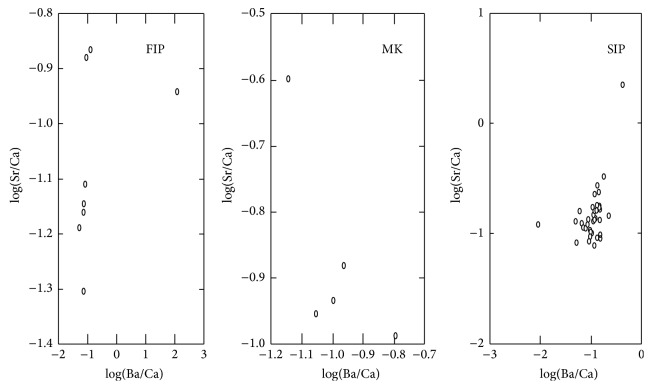
Bivariate plot of log(Ba/Ca) and log(Sr/Ca) for the three epochs.

The correlation coefficients of log(Ba/Ca) and log(Sr/Ca) for the FIP, the MK, and the SIP were 0.403, 0.721, and 0.486 for the three epochs, respectively; this positive significant correlation between Ba/Ca and Sr/Ca (Figure 1) indicates that they reflect intact biological values.

### 3.3. Determination of Strontium Concentrations in the Three Epochs

**Figure 2:**
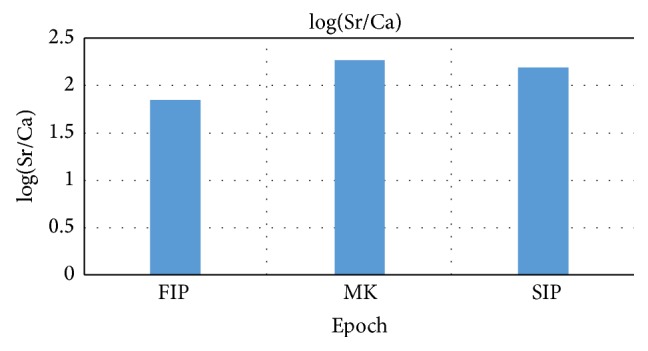
Comparison of log(Sr/Ca) for the three epochs under investigation.

Using the strontium calibration curve created by Samek et al. [21] strontium concentration relative to calcium (Sr/Ca ratio) was obtained in ppm for the three historical epochs (FIP, MK, and SIP). Results of ANOVA test comparing the log(Sr/Ca) between the 3 historical eras revealed that there is a significant difference between the three epochs (*P* = 0.002). Post hoc comparisons LSD test indicated that the log(Sr/Ca) of the FIP was significantly lower than that of both the MK and the SIP as shown in Table 3 and Figure 2.

## 4. Discussion

### 4.1. Discussion of Methodology

The studies concerning elemental analysis of ancient Egyptians archeological bones are very rare. This may be attributed to the higher anthropologists' interest in more classic studies such as anthropometrical and pathological researches. The published anthropological studies concerning the skeletal collection of the Aswan population had covered many domains except diet reconstruction through elemental analysis of bones; that is why it was recommended by Rösing [34] to conduct researches concerning this item.

Benefiting from LIBS technique as a quasinondestructive technique the multielemental analysis of archeological mandibles belonging to Aswan population and dated to FIP, MK, and SIP was performed without the destruction of the mandibles that are considered as “precious” bones as the mandible represents a part of the skull.

The analysis of mandibles was restricted to the mandibular body which is considered as one of the sites containing the highest mineralized regions in the mandible [35]. It was chosen to use the cortical bone of mandibles because the high mineralization of the cortical bone and its small surface area in comparison to spongy bone render it more resistant to the diagenetic processes [36] and also because the skull cortical bone remodeling rate is slower than that in spongy bones [37]. During remodeling trace elements can be removed or deposited from bone apatite [38].

The cortical bone elemental analysis provides data concerning the last 6–10 years of the individual life [39], as the cortical bone is characterized by a lower biological activity than trabecular bone and it can be represented as the site of elemental storage, while the spongy bone is characterized by a greater biological activity and greater turnover rate because it is closest to bone marrow and blood plasma and hence a more rapid metabolism is available [38].

In the current study only mandibles were analyzed, as it is recommended according to Grupe [36], to use compact bone from corresponding anatomical site in the studies of paleodiet reconstruction. That is because the rate of turnover of compact bone may differ in the same individual according to the anatomical location [40].

Only adult individuals were included in this work as the bones of immature individuals are more susceptible to diagenetic changes as they are characterized by their thinner compact bone and lower level of mineralization in comparison to the bones of adult individuals [41].

### 4.2. Diagenesis Evaluation

Diagenetic alterations can change the elemental content of archeological bones both by physical contamination and by chemical reaction [6].

In order to evaluate the extent of diagenesis the measurement of soil pH was conducted. The soil in Qubbet el Hawa is nearly neutral. The neutrality of the soil may be considered as an indicator of the good preservation state of bones [42]. Thanks to the ancient Egyptians burial customs the stability of the burial conditions over years is enhanced by the dryness of tomb environment as generally ancient Egyptians buried their dead in arid areas.

But, according to Nicholson [43], “*The pH alone is insufficient as a predictor of skeletal preservation,*” so the elemental content of soil was determined and compared to that of bones.

The significant higher levels of Sr and Ca in bones in comparison to that in soil was considered as an indication that Sr and Ca levels represent the biological levels, but the main argument against diagenesis was the results showed by the significant correlation between the Sr/Ca ratio and the Ba/Ca ratio that indicate that these ratios reflect the biological values. This test was used not only for its effectiveness over classic tests [33] but also because the target of analysis was the mandible which represents a “precious” bone because it is a part of the skull, so the use of any destructive method for assessing the extent of diagenesis was avoided.

### 4.3. Diet Reconstruction

Many studies used the Sr/Ca ratio as a quantitative indication of the plant to meat ratio basing on the biopurification of strontium through the food chain, for example, that of Schoeninger and Peebles [20], El-Tayeb [24]; however, this way of interpretation had been criticized because the relationship between bone Sr/Ca and the plant/meat ratio is not linear, also, as bone Sr/Ca ratio is negatively affected by calcium-rich food consumption, the low bone Sr/Ca ratio is not necessarily the result of reliance on meat [44].

It is important to note that ethanol consumption causes a decrease in bone strontium and barium, while a low-protein diet accompanied by ethanol consumption increases the bone strontium and decreases that of barium [45]. Also, ways of cooking may affect Sr content of food [46]. In addition, it is indicated by Katzenberg [47] that the concentration of strontium in plant tissues depends on many factors such as the soil type.

Before discussing the results it is important to note that differences in the Sr/Ca ratio between the three groups could not be attributed to alcoholism, as, in contrast to beer, wine was available mainly for the higher social class [48]. In addition, prohibition of some sorts of food is not expected as taboos concerning eating some sorts of food in the ancient Egyptian culture were very few [49]. Also, as little is known about ancient Egypt methods of cooking [50], it is impossible to conclude to what extent ancient Egyptian processing could affect strontium levels in foods.

That log(Sr/Ca) ratio in case of the FIP is significantly lower than that of MK and SIP. The FIP was a period accompanied by the rise of nomarchs authority and the dissolution of the central government: it was the period of feudalism [51]. According to Welc and Marks [52], at the end of the Old Kingdom, failure of the rains over the Ethiopian Highlands led into the failure of Nile floods. According to Bell [53], this situation that extended for decades during the FIP was the “*crisis that shattered a weakened central government utterly unable to cope with the problem, and decimated the Egyptian people*.” As explained by Erman [54], there is an indication in the text named “*Admonitions of an Egyptian Sage*” that famine pushed the people to eat which they used to feed to the domesticated birds and mammals. Then, the introduction of “unusual” sorts of foods or plants in the diet of Elephantine nobles followers and descendants during years of famine is suggested by this study. The high calcium or very low strontium contents of these newly and “exceptionally” introduced food types may be the cause of low Sr/Ca ratio relative to that in following eras, the MK and the SIP.

In addition, as indicated by Vandier [55] one of the adopted strategies against famine in ancient Egypt was the loans of cereals between nomes, for example, Ankhtifi, the nomarch of “Edfu” and “Hierakonpolis” during the early FIP supported the neighboring cities, including Elephantine, during years of famine, as he described in his tomb. Thus, it is expected that the consumption of cereals-staple food-exported from other nomes, that is, from regions with different chemical soil composition, caused this low level of Sr/Ca during years of famine where Aswan population cannot be considered as self-sufficient.

During the MK, the whole situation had been changed: Egypt was reunified and feudalism disappeared [51]. The social revolution during the FIP led to radical changes in moral values that led in turn to equal opportunities availability. Thus, the rise of a new class of officials that were proud to be self-made was allowed [56], and the flourishment of the ancient Egyptian middle class [51] that was “safeguarded from famine” [57] took place. The current study suggested that considering the amelioration of conditions, especially for the middle class to which the population under investigation is belonging, it is expected that dietary habits of Elephantine nobles followers and descendants had been differed from that of the FIP. The typical Dynastic diet consumption was adopted with no need for the consumption of “exceptional” sorts of food nor cereals import.

The Sr/Ca ratio of the group of the SIP is nearly equal to that of the MK. According to Ryholt [58] famines struck Egypt during the reign of the two competing Dynasties, the 13th and the 14th ones, as well as during the reign of the 16th Dynasty that governed the south of Upper Egypt. Although the anarchy swept all over the country during the SIP, the dissolution of the central government did not appear as the feudalism disappeared [51]. According to Abu-Taleb [59], by the disappearance of feudalism during the MK and the SIP, the individualism associated with state socialism flourished. That means that although the SIP was a period of weakness, general conditions were different from that of the FIP which is reflected in a Sr/Ca ratio that is nearly similar to that of the MK: despite the anarchy and famines, the calcium sources of the population under investigation remained the same during the MK and the SIP which may reflect an indication that food intake during the SIP in Elephantine was not hardly affected to the same extent of the FIP, taking in mind that as indicated by Vandier [55] few texts concerning famine during the SIP are available. One of these texts reflects the amelioration of political and economical conditions at the end of the SIP during the reign of the 17th Dynasty in Upper Egypt to the extent that the legitimate authority in the South was ready to struggle the Asiatics that ruled Lower Egypt (The Hyksos). In Carnarvon Tablet, the pharaoh Kamose recorded his discussion with the counselors about the situation in Egypt where they said “*We are tranquil in our part of Egypt. Elephantine is strong, and the middle part (of the land) is with us as far as Cusae. Men till for us the finest of their lands. Our cattle pasture in the Papyrus marshes. Corn is sent for our swine. Our cattle are not taken away… He holds the land of the Asiatics; we hold Egypt*” (quotation from Gardiner [60]).

It is recommended to conduct isotopic studies on the Aswan population to achieve more detailed interpretations about food customs of this area during ancient epochs. This will be completed with detailed paleoclimatological, zooarcheological, archeobotanical, and land use studies.

## Figures and Tables

**Table 1:** Number of mandibles for each epoch.

Grave number	Sample size	Era
26	17	FIP
89	19	MK
88	33	SIP

**Table 2:** Comparison between soil and bone elemental relative intensities for the three epochs under investigation.

	Soil	Bone		Diff.	*t*-value	*P* value
		Mean	SD			
FIP						
Ca	3.31	10.108	4.241	6.798	6.61	*P* = <0.001^*∗*^
Sr	0.44	0.830	0.406	0.3901	3.958	*P* = <0.001^*∗*^
Ba	2.14	0.831	0.37	−1.309	−14.581	*P* = <0.001^*∗*^
MK						
Ca	3.31	56.126	26.577	52.816	8.662	*P* = <0.001^*∗*^
Sr	0.44	9.351	8.668	8.911	4.481	*P* = <0.001^*∗*^
Ba	2.14	5.687	1.876	3.547	8.24	*P* = <0.001^*∗*^
SIP						
Ca	3.31	17.551	23.244	14.241	3.52	*P* = <0.001^*∗*^
Sr	0.44	2.641	4.578	2.201	2.762	*P* = 0.007^*∗*^
Ba	2.14	1.676	2.163	−0.464	−1.231	*P* = 0.223

SD: standard deviation.

^*∗*^Significant at *P* < 0.05; *P* > 0.05 (nonsignificant), *P* < 0.05 (significant), and *P* < 0.01 (highly significant).

**Table 3:** Strontium concentration and log⁡(Sr/Ca) in mandibular bodies belonging to the three epochs.

Era	Sr (ppm)	log⁡(Sr/Ca)
FIP	71.4^a^	1.85^a^
MK	187.5^b^	2.272^b^
SIP	156.7^b^	2.1948^b^

Different small letters indicate significant difference between different age groups according to Tukey LSD pairwise comparison.
